# Study protocol of a cluster randomized controlled trial of strategies to increase antenatal iron and folic acid supplementation and malaria prophylaxis in rural south-central Côte d’Ivoire

**DOI:** 10.1186/s12889-020-09626-0

**Published:** 2020-10-27

**Authors:** Siaka Koné, Jürg Utzinger, Nicole Probst-Hensch, Daouda Dao, Günther Fink

**Affiliations:** 1 Centre Suisse de Recherches Scientifiques en Côte d’Ivoires, 01 BP 1303 Abidjan 01, Côte d’Ivoire; 2 Swiss Tropical and Public Health Institute, Basel, Switzerland; 3 University of Basel, Basel, Switzerland

**Keywords:** Antenatal iron and folic acid supplementation, Malaria chemoprophylaxis, Maternal and child health, Cluster randomized controlled trial, Health and demographic surveillance system, Cost-effectiveness

## Abstract

**Background:**

Coverage of antenatal iron and folic acid supplementation (IFAS) and intermittent preventive treatment of malaria in pregnancy (IPTp) remains low in many countries. Evidence on the most effective ways to increase both IFASIPTp is mixed overall, with only few studies directly identifying cost-effective ways to increase coverage of both interventions. The proposed study aims to assess the cost, impact and relative cost-effectiveness of two complementary strategies of increasing IFAS and malaria chemoprophylaxis coverage among pregnant women relative to the current default system in a rural low-income setting of sub-Saharan Africa.

**Methods/design:**

This study will be carried out in the Taabo health and demographic surveillance system (HDSS) in south-central Côte d’Ivoire. This is a cluster-randomized trial targeting 720 consenting pregnant women aged ≥15 years. The 118 clusters constituting the Taabo HDSS monitoring area will be randomly allocated to one of the following three groups with equal probability: a control group, an information only group, and an information plus home delivery group. To assess the relative effectiveness of each strategy, we will conduct an endline survey within the first 2 weeks after delivery. The primary outcomes of the trial will be maternal post-partum anaemia and malaria infection. Anaemia will be assessed using HEMOCUE devices; malaria infections will be assessed using standard rapid diagnostic tests named CareStart™ Malaria Pf (HRP2) Ag RDT (Multi Kit with capped lancet and inverted cup specimen transfer device). Other outcomes will include self-reported adherence to supplementation and malaria chemoprophylaxis, as well as miscarriages, stillbirths and low birth weight deliveries.

**Discussion:**

This study will assess the cost-effectiveness of two alternative strategies to increase antenatal IFAS and malaria chemoprophylaxis coverage among pregnant women in rural Côte d’Ivoire and similar settings.

**Trial registration:**

ClinicalTrials.govNCT04250428; Registered 31 January 2020.

## Background

Despite major progress in access to antenatal care over the past years, coverage of essential antenatal interventions remains limited in many low- and middle-income countries (LMICs) [1]. At the same time, prevalence of maternal anaemia [2, 3] and exposure to malaria in pregnancy remain high [4]. In Côte d’Ivoire, 59% of pregnant women are estimated to be anaemic (haemoglobin< 110 g/L), only a minority of women receive antenatal iron supplementation consistently throughout their pregnancy, and less than one third of women receive the recommended three doses of intermittent preventive treatment of malaria in pregnancy (IPTp) [5–7]. Iron supplmentation and malaria chemoprophylaxis have been shown to be highly effective for reducing the risk of stillbirth, prematurity and low birth weight, and have been highlighted as essential for reducing the burden of malnutrition in the 2013 Lancet series [8].

Low coverage rates of essential antenatal care interventions have been attributed to lacking demand from beneficiaries (e.g. low antenatal care attendance), infrequent early health system contact with women, limited funding, stock outages and ineffective management of supplies [8–11].

In general, limited coverage of essential health services as well as limited adherence to national protocol have been attributed to multiple challenges in the health system. These challenges include the lack of knowledge on importance of medication, interrupted supply and stock outs, high cost of care, lack of availability of services, and demand-side barriers such as distance, education, opportunity cost, and cultural and social barriers [12–15]. To address these challenges successfully, a large number of community-based studies assessed a range of interventions, including community-based distribution of drugs, vaccines, or other public health services. In 2018, a systematic review of strategies identified 28 studies evaluating the treatment coverage in community-based public health programmes [16]. These studies covered a range of different strategies, including community-based treatment, distributor incentives, distribution along kinship networks, intensified information, education, and communication activities, fixed-point delivery, conversion from school- to community-based delivery, and management by a non-governmental organization. Services delivered included community-based public child health programmes such as vitamin A supplementation, child immunizations, and mass drug administration campaigns targeting neglected tropical diseases. The largest positive influence on treatment coverage was found for four strategies: community-directed distribution, incentives to increase distributor motivation, distribution along kinship networks and implementation of intensified information, education and communication activities. A 2016 Cochrane review evaluated the effectiveness of community-based health education and household monetary incentives in child immunization coverage in LMICs. Overall, health education at village meetings or at home, as well as household monetary incentives had little or no effect on full immunization coverage [17]. Vouchers have also been widely used to promote maternal and newborn health in LMICs: a systematic review based on seven previously published systematic reviews [18] found that reproductive health voucher programmes increased utilization of reproductive health services, improved quality of care, and improved population health outcomes. In another Cochrane review focusing on antenatal care, 34 randomized controlled trials testing community-based interventions to improve uptake of antenatal care (media campaigns, education or financial incentives for pregnant women), and health systems interventions (home visits for pregnant women or equipment for clinics) were identified. The review highlighted several potentially effective interventions, and suggested a combination of interventions for stronger impact [19].

Overall, evidence on the most effective ways to increase both IPTp and antenatalIFAS remains mixed,with very few studies directly identifying cost-effective ways to increase coverage of both interventions. 

The objective of the proposed research is to assess both the cost, effectiveness and relative cost-effectiveness of two of the most commonly used strategies in a rural low-income setting compared to the default system. Our main hypothesis is that uptake of preventive antenatal services can be improved by both demand and supply strategies, but that supply side interventions are more cost-effective and equitable than demand side interventions despite their higher relative cost. The results of this study will help to identify the most effective ways to increase antenatal IFAS and IPTp coverage among pregnant women in rural Côte d’Ivoire and similar settings.

## Methods/design

### Study design

The main idea of this study is to test and compare the relative effectiveness and cost-effectiveness of demand and supply side interventions encouraging preventive care use through a single trial. Secondary objectives include determining programme impact (i) on the prevalence of post-partum anaemia and malaria infection, and (ii) on children’s birthweight.

**Fig. 1:**
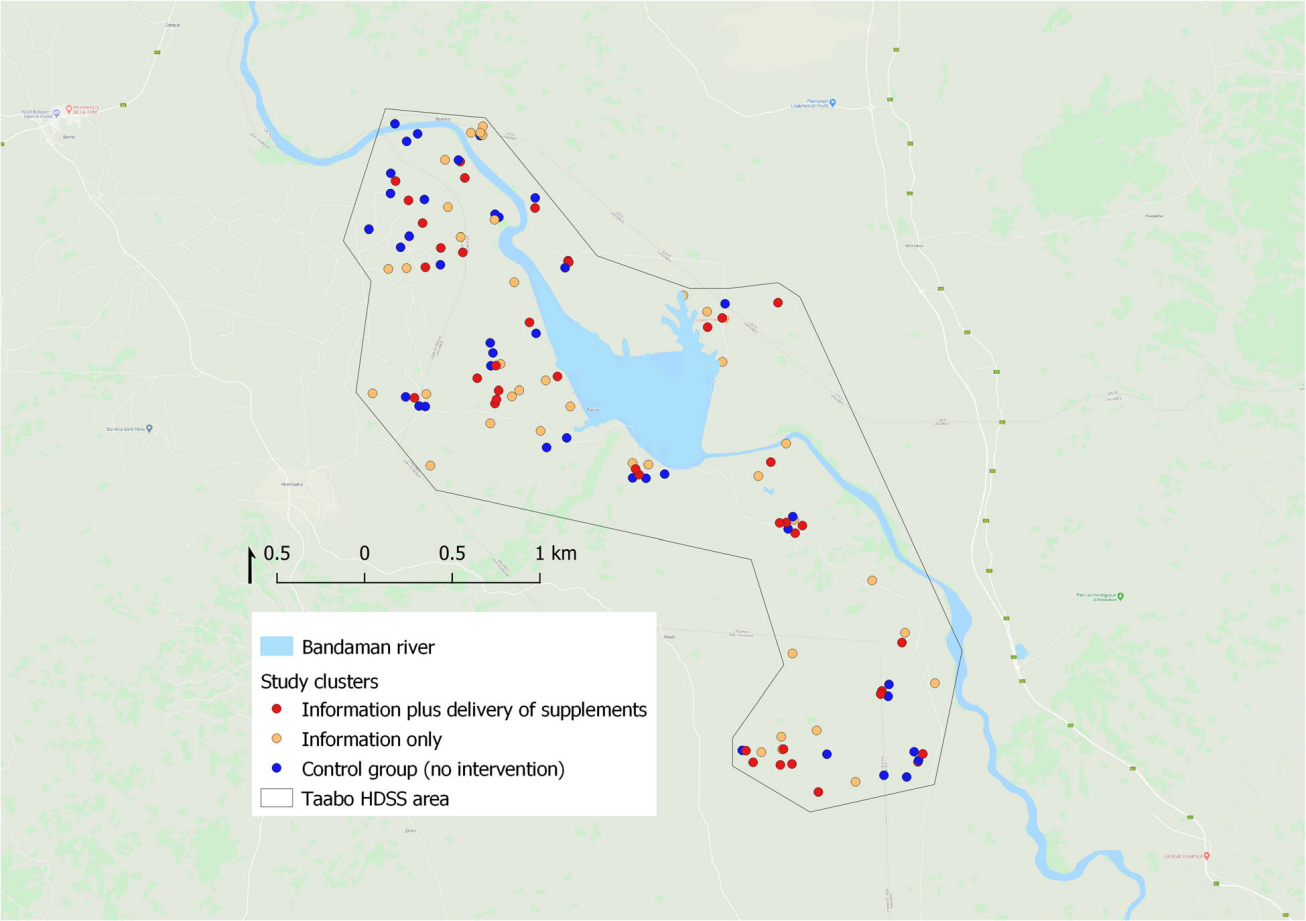
Map of the study area and clusters. Notes: The map was generated using QGIS geospatial database software version 3.4.4. Administrative boundary data and cluster centroids were collected through the HDSS. The base background layer showing roads, rivers and lakes was extracted from google earth using QGIS

The study will be designed as a cluster-randomized experiment with three parallel arms: a control arm, an information only or “demand generation” arm, and an information plus direct distribution arm. We expected to recruit approximately 240 pregnant women for each arm, for a total sample size of 720 women. This study will be carried out in the Taabo health and demographic surveillance system (HDSS) in south-central Côte d’Ivoire. The Taabo HDSS has continuously monitored a population of approximately 45,000 since 2009. The pilot study will target 720 pregnant women across the 118 neighbourhoods within the HDSS monitoring area. Each cluster corresponds to a neighbourhood (“quartier”) in larger villages, or a smaller separate settlement (“hamlet”). The 118 clusters will be split into three groups: a control group (39 clusters), an information group (39 clusters), and a distribution of free supplements group (40 clusters) (Fig. 1).

**Fig. 2:**
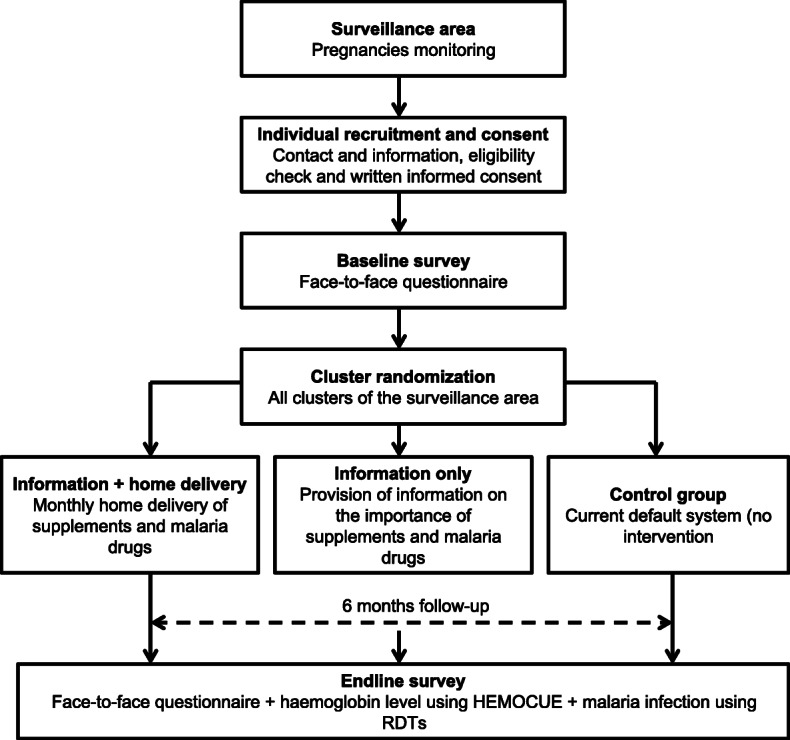
Study overview

A study overview showing the main components and timeline is given in Fig. 2.

### Setting

The proposed research will be implemented in the Taabo HDSS area in the sub-prefecture of Taabo. The HDSS covers an area of 980 km_2_ and comprises one small town (Taabo-Cité), 13 villages and over 100 smaller hamlets attached to the same villages [20]. In terms of health infrastructure, the HDSS features one general hospital and 10 health centres. Population surveillance is implemented three times per year and focuses on pregnancies, births, deaths and migration of residents [20, 21]. For all deaths reported, verbal autopsies (VAs) are conducted using WHO VA instrument to establish likely causes of death [22–25].

Data collected between 2009 and 2011 highlights the high prevalence of communicable diseases such as malaria, acute respiratory infections, HIV/AIDS and pulmonary tuberculosis [24]. In 2018, 1436 pregnancies were reported in the Taabo HDSS. 73% of the local population are Ivorians (predominantly Akans); most non-Ivorians are from Burkina Faso [20].

### Selection of study participants

#### Recruitment

Pregnant women in the first or second trimester of their pregnancy, living in the Taabo HDSS with an age ≥ 15 and ≤ 49 years will be eligible to participate in this study. Gestational length will be assessed based on self-reported date of conception or date of last menstrual period. Key informants will be recruited by the study team in all areas to report pregnancies as early as possible to the study staff. Key informants will be given a reward of 1000 CFA (approximately USD 2) for each pregnancy reported. Once the study coordinator has been informed about a new pregnancy, a local field enumerator will be sent out to invite pregnant women to participate in the study. Conditional on her consent, the women will be automatically assigned to a group based on her residence and a short baseline questionnaire will be conducted.

#### Participant information and consent

We will use two separate informed consent forms for this study. A general study participation consent form that will primarily cover the baseline and endline surveys, and consent forms for the two interventions. Mothers in the information (intervention) arm will be informed about the objective of this intervention. Mothers in the supplements arm will be informed about the objective of the arm as well as the package of this intervention. These intervention consent forms will be short and administered by study staff at the beginning of their first visit.

For mothers under the age of 18 years, permission to participate in the study will be sought from a legal representative in the household. For illiterate mothers, we will use a thumbprint instead of a signature on the consent form. Participants may decide not to continue participating in the study at any time for any reason if they wish to do so without any particular involvement. The investigator may decide to remove a participant from the study for reasons of ethical concerns or insufficient participant recruitment.

### Intervention

As part of this study, we will test two interventions: an information only intervention and an information plus delivery intervention.

The information only intervention focuses on empowering pregnant mothers to adopt appropriate health practices and health behaviour. Health staff (midwives and nurses) will visit all women in this arm at the beginning of their pregnancy (after baseline) and inform women regarding the importance of antenatal IFAS as well as IPTp. Specifically, the nurse will talk with participating women about the benefits, adverse events, and when, why, and how to take IFAS and sulfadoxine-pyrimethamine (SP). The nurse will also provide pregnant women with a phone number they can call in case they have questions related to antenatal care, supplements or IPTp, and will encourage women to attend antenatal care in accordance with national guidelines.

In the information plus delivery arm, women will receive a similar first visit by a study nurse, followed by monthly home visits. During the initial visit, study staff will inform women about recommended antenatal care attendance and supplementation and inquire about supplements received. All women not having received supplements from their antenatal care visits will be then be directly provided with a monthly supply of iron and folic acid. Similarly, all women in their second or third trimester not having received SP will be directly provided SP together with additional information and instructions on how to take the medication each month. At the end of each monthly visit, study staff will remind women about their next antenatal care appointment and remind them to take supplements daily.

### Data collection

Table 1 presents a summary of all measurements conducted in the study. Data will be collected by a trained researcher team. In addition to the baseline collection, we will collect detailed monitoring data on home visits as well as cost data for the two arms. To assess the relative effectiveness of each strategy, we will conduct an endline survey within the first 2 weeks after delivery. The form to be used to conduct the endline survey among women will combine both information on prenatal supplementation and IPTp (supplements, doses, period, frequency, and date, anaemia and malaria status), socio-demographic characteristics e.g. (maternal age, education attainment and household assets), and pregnancy outcome (e.g. live birth, still birth, preterm birth and birth weight,). Given that we expect women’s self-report on antenatal IFAS not be very accurate, we will collect haemoglobin level and malaria infection prevalence as primary study outcome measures at endline. Haemoglobin levels will be assessed using HEMOCUE devices; malaria infection will be tested using standard rapid diagnostic tests (RDTs). All endline surveys will be conducted by study nurses who are familiar with both test procedures.

**Table 1:** Overview of study outcomes and measurements

	Baseline	Endline (6 months)
**Household’s characteristics**		
Primary source of drinking water	X	
Source of lighting	X	
Materials and equipment	X	
**Woman’s characteristics**		
Date of birth	X	
Marital status	X	
Level of education	X	
Pregnancy duration	X	
Number of live births	X	
Total number of pregnancies	X	
ANC attendance	X	X
Gestational age at first ANC visit	X	
**Pregnancy related information after delivery**		
Pregnancy outcome		X
Child’s sex		X
Birth weight		X
Place of delivery		X
Assistance during delivery		X
Delivery process		X
Delivery cost		X
Performed tests during ANC		X
Antenatal IFAS uptake (number, dose)		X
Malaria prevention (number, dose)		X
Adverse events from iron uptake		X
Haemoglobin level		X
Malaria infection		X

*ANC* antenatal care; *IFAS* iron and folic acid supplements

### Data handling

The interview data will be collected by trained field enumerators using portable electronic devices (tablets) with incorporated data entry forms. Open Data Kit (ODK) will be used for entering data. The master database will be managed in a secure server. HEMOCUE devices and standard RTD results linked to unique numbers will be recorded in lab sheets and entered into a database.

Project data will be handled with utmost discretion and will only be accessible to authorized personnel who require the data to fulfil their duties within the scope of the research project. To ensure confidentiality, in the questionnaire and lab forms, participants are only identified by a unique participant number, which will be anonymized and cannot be traced back to the person without a separate ID key.

We will prevent the problem of missing data by well planning the study and collecting the data carefully. In case of missing data, we will use multiple imputation to replace the missing values with a set of plausible values that contain the natural variability and uncertainty of the right values.

To maintain data security and prevent unauthorized access, the data server will be restricted with a security password, and access will be given only to selected persons. A backup of the data file will be done periodically and kept in a different place. Following the study, the data will be archived in location and period in accordance with the local regulations.

This project is part of the Taabo HDSS, which is affiliated to the Centre Suisse de Recherches Scientifiques en Côte d’Ivoire and the Swiss Tropical and Public Health Institute in Switzerland. The data resulting from this project will therefore be shared on both the Centre Suisse de Recherches Scientifiques and the Swiss Tropical and Public Health Institute servers. The codebook document will contain information on study design, sampling methodology, fieldwork, variable-level detail, and all information necessary for a secondary analyst to use the data accurately and effectively.

All data collected as part of this project will be made publicly available after publication of the main study results.

### Sample determination

The unit of randomization will be neighborhoods, locally referred to as “quartiers” or “hamlets”. The study is powered to detect a 20%pt. decline in maternal anaemia with power 0.8 and alpha 0.05, assuming a control group anaemia prevalence of 50%, 6 women per cluster, and an intra-class correlation (ICC) of 0.25. The ICC estimate was based on a previous analysis of antenatal care access patterns in the Taabo HDSS. Assuming a follow-up rate of 90%, this requires an initial enrolment of 240 women per arm, or a sample of 720 women in total. Given the births rates observed in the past 3 years, we anticipate to enroll these 720 women over a 6-month period. Based on the latest Demographic and Health Survey conducted in the region, we expect 21% of women in the control group to be malaria positive. In order to achieve power 0.8, we would need a two third reduction in RDT positivity after birth with the intervention.

### Randomization procedures

Neighbourhoods were randomized to the three groups using min-max randomization [26]. Specifically, 100 random allocation were created using the Stata 15 SE software package. For each random draw, the three groups were compared with respect to the average number of births and average antenatal care attendance in the 2016–2018 period. The random draw with the smallest differences between the three groups (largest minimum *p*-value) was used for final treatment assignment.

The group assignment of women will only be known to the study coordinator, who will directly manage all interventions.

### Blinding procedures

Given that we wish to study the best delivery of interventions, we cannot blind subjects to treatment. Treatment status will, however, not be revealed to the survey team conducting the endline assessment. Un-blinding of subjects will not be necessary.

### Statistical analyses

The health outcomes to be studied are post-partum anaemia, malaria infection, and birth weight. Post-partum anaemia will be defined as a haemoglobin concentration less than 11 g/dL and malaria infection will be defined by a positive result of the RDT. Logistic regression models will be used to compare post-partum anaemia and malaria infection in the two intervention groups to the control group. For birth weight as well as haemoglobin levels as continuous variable, we will use basic linear regression models. Generalized estimating equations with robust variance-covariance estimation will be used to account for clustering [27, 28]. The basic regressions models will compare outcomes in treated and control women, with standard error corrections for grouped data. In adjusted estimates, we will control for basic maternal covariates such as age, education, and residence. The power calculations were done as two-group comparisons, allowing to estimate differences between either of the two intervention groups and the control group. Additional subgroup analysis focusing only on a dose-response analysis will also be conducted to estimate average treatment effects on the treated.

### Access to care during and after the trial and compensation

All subjects will have access to public antenatal and post-natal care during and after the trial. Women in both treatment and control groups with diagnosed malaria infections and haemoglobin levels below 11 g/dl will be referred to the nearest health centre for further testing and treatment. No compensation will be provided to study subjects.

### Cost and cost-effectiveness

Throughout the trial, detailed cost data for implementing both intervention arms will be collected. The data will be used to compare the observed increases in supplementation coverage to the cost of each arm. Incremental cost effectiveness ratios will be defined as additional cost per pregnant woman divided by the observed increase in the probability of receiving appropriate ANC (IPTp + IFAS).

## Discussion

This paper describes the design of a cluster-randomized study that will evaluate the effectiveness and cost-effectiveness of two alternative strategies in increasing IFAS and malaria chemoprophylaxis coverage among pregnant women relative to the current default system. To our knowledge, this is the first study in Côte d’Ivoire using data stem from an HDSS, which is a well-characterized population-based cohort that is subject to longitudinal surveillance. Results from this study will help in identifying the most effective and cost-effective strategy to increase antenatal IFAS and IPTp coverage in rural Côte d’Ivoire and similar settings. The results of the study will be shared through a peer-reviewed article as well as through the communication offices at the Centre Suisse de Recherches Scientifiques and the Swiss Tropical and Public Health Institute.

The choice of interventions is made not only based on the results of past studies [16–18] but also on the priority of national challenges in maternal and child health. The latest data from the Taabo HDSS suggests that prenatal supplementation with iron and folic acid covers less than 50% of pregnant women. IPTp has been adopted since 2005. However, the effectiveness of protecting the mothers and unborn child from adverse events from malaria depend on the rigorous adherence of this policy, coverage, and the number of ANC visits of the expectant mothers [29, 30].

The implementation of this project into the Taabo HDSS will make recruitment of the participants easy. The project will also be conducted in close collaboration between the Centre Suisse de Recherches Scientifiques, Swiss Tropical and Public Health Institute, local physicians and nurses, and communities leaders based on mutual agreement with local authorities from the whole HDSS’s area to guarantee smooth implementation. The Taabo HDSS platform reduces the risk for loss to follow-up since the fieldwork teams hold close contact with the local population.

Although the study is designed as a cluster-randomized controlled trial, contamination between hamlets or neighbourhoods cannot be excluded. In cases where clusters correspond to hamlets, the next settlement is typically at least 1 km away. For urban neighbourhoods, distances are much smaller, with households on the opposite side of the street receiving opposite treatments in some cases. For the direct delivery arm, spillovers seem somewhat unlikely, because the study team will simply not deliver the same intervention to non-treated women. For the information only treatment, spillovers are certainly possible, and would bias the estimated impacts towards zero. We will explore such contamination effects through spatial analysis in our sensitivity checks.

The project team may also face some push-back or complaints regarding adverse events related to iron consumption. We will make subjects aware of this risk in the informed consent form and use the initial information session to further prepare pregnant women for this. We will also try to minimize these adverse events by asking mothers who have complaints to take supplements after major meals. Another problem is that some women may not attend ANC if they receive supplements at home. ANC attendance is critical for preparing women for delivery, for understanding warming signs during pregnancy and childbirth, and for the prevention of pregnancy-related morbidity factors. To ensure attendance to ANC, all women will be reminded to attend the scheduled visits during the first visit in both arms, and during each of the visits by our study team in the home delivery arm.

Last, there are some concerns that folic acid supplementation may increase the rate of treatment failures with SP [31]. Given that national protocols demand both iron and folic acid supplements, we cannot investigate this question experimentally in our study – post-hoc analysis of relative SP efficacy among women taking and not taking iron and folic acid supplements may however be possible.

## Data Availability

The data collected as part of this trial will be made available to researchers upon publication of the main results.

## Abbreviations

IPTp: Intermittent preventive treatment of malaria in pregnancy
HDSS: Health and demographic surveillance system
LMICs: Low- and middle-income countries
SP: Sulfadoxine-pyrimethamine
